# Ultrafine carbon particles down-regulate CYP1B1 expression in human monocytes

**DOI:** 10.1186/1743-8977-6-27

**Published:** 2009-10-16

**Authors:** Christiane Eder, Marion Frankenberger, Franz Stanzel, Albrecht Seidel, Karl-Werner Schramm, Loems Ziegler-Heitbrock, Thomas PJ Hofer

**Affiliations:** 1 Helmholtz Zentrum Muenchen, German Research Center for Environmental Health, Clinical Cooperation Group Inflammatory Lung Diseases, Institute of Lung Biology and Disease and Asklepios Fachkliniken Muenchen-Gauting, Robert-Koch-Allee 29, 82131 Gauting, Germany; 2 Asklepios Fachkliniken München-Gauting, Robert-Koch-Allee 2, 82131 Gauting, Germany; 3 Biochemisches Institut für Umweltcarcinogene, Lurup 4, 22927 Grosshansdorf, Germany; 4 Helmholtz Zentrum Muenchen, German Research Center for Environmental Health, Institute for Ecological Chemistry, Ingolstaedter Landstrasse 1, 85764 Neuherberg, Germany; 5 Current address: Lungenklinik Hemer, Theo-Funccius-Strasse 1, 58675 Hemer, Germany

## Abstract

**Background:**

*Cytochrome P450 monoxygenases *play an important role in the defence against inhaled toxic compounds and in metabolizing a wide range of xenobiotics and environmental contaminants. In ambient aerosol the ultrafine particle fraction which penetrates deeply into the lungs is considered to be a major factor for adverse health effects. The cells mainly affected by inhaled particles are lung epithelial cells and cells of the monocyte/macrophage lineage.

**Results:**

In this study we have analyzed the effect of a mixture of fine TiO_2 _and ultrafine carbon black Printex 90 particles (P90) on the expression of *cytochrome P450 1B1 *(*CYP1B1*) in human monocytes, macrophages, bronchial epithelial cells and epithelial cell lines. *CYP1B1 *expression is strongly down-regulated by P90 in monocytes with a maximum after P90 treatment for 3 h while fine and ultrafine TiO_2 _had no effect. *CYP1B1 *was down-regulated up to 130-fold and in addition *CYP1A1 *mRNA was decreased 13-fold. In vitro generated monocyte-derived macrophages (MDM), epithelial cell lines, and primary bronchial epithelial cells also showed reduced *CYP1B1 *mRNA levels. Benzo[*a*]pyrene (BaP) is inducing *CYB1B1 *but ultrafine P90 can still down-regulate gene expression at 0.1 μM of BaP. The P90-induced reduction of *CYP1B1 *was also demonstrated at the protein level using Western blot analysis.

**Conclusion:**

These data suggest that the P90-induced reduction of CYP gene expression may interfere with the activation and/or detoxification capabilities of inhaled toxic compounds.

## Background

Several epidemiologic studies attribute increased morbidity and mortality to exposure to environmental particles [1-3]. These adverse health effects due to the inhalation of particulate matter are a topic of ongoing scientific and public concern. Particulate matter (PM) is a complex mixture of many different components, which can be characterized by origin (anthropogenic or geogenic), by physicochemical properties (such as solubility) or by particle size. Particles with a mean aerodynamic size between 10 and 2.5 μm (PM_10_) are classified as coarse particles, fine particles have a size between 2.5 and 0.1 μm and particles with a diameter less than 0.1 μm are termed ultrafine. Not only the particle size but other particle-associated parameters like particle number, surface area or reactive compounds adsorbed to the surface may be involved in the observed health effects [4]. Because of their small size, ultrafine particles contribute only modestly to total mass, but they are the predominant fraction by number in PM. Most urban particles result from combustion processes, therefore the major fraction contains ultrafine carbonaceous particles [5]. After deposition in the lung larger particles are phagocytized by alveolar and airway macrophages [6,7], but the fine and ultrafine carbon particles remain in the lung for a longer period of time [5]. Ultrafine particles are phagocytized to a minor extend but they can still enter macrophages and epithelial cells and even penetrate into the circulation. Thus ultrafine particles not only trigger local inflammatory reactions in the lung but also cause systemic extrapulmonary effects [8]. Ultrafine particles also have the capacity to inhibit phagocytosis by alveolar macrophages [9]. Macrophages and their monocyte progenitors are major elements of the inflammatory response. In addition to performing phagocytosis they can release inflammatory mediators such as cytokines and chemokines and they are crucially involved in destruction of microbes and particles using various enzymatic systems [10]. Cytochromes like *CYP1B1 *are also expressed by macrophages and these enzymes are part of the "digestive" and detoxifying machinery of these cells [8].

The xenobiotic metabolism can be divided into two phases: modification (phase I) and conjugation (phase II). An important group of phase I enzymes consists of the *cytochrome P450 oxidases (CYP) *which belong to the monoxygenases. In humans 57 *CYPs *are known and about 25% of them are considered to be involved primarily in the xenobiotic metabolisms [11]. Superfamily members are classified according to the similarity of their primary structure. The expression of the *CYP1 *subfamily can be induced by polycyclic aromatic hydrocarbons (PAH), which are ubiquitously occuring environmental carcinogens [12] and are particularly known to be present in cigarette smoke [13]. The induction of *CYP1 *genes is regulated by a heterodimer of the aryl hydrocarbon receptor (AhR) and the aryl hydrocarbon receptor nuclear translocator (Arnt) [14]. Two cytochromes, *CYP1A1 *and *CYP1B1*, are mainly involved in the formation of ultimate carcinogenic diol-epoxides of PAH such as benzo[*a*]pyrene (BaP) [15]. The expression of these enzymes is largely extrahepatic and both enzymes are present in many tumor tissues [16,17]. *CYP1B1 *has been identified as a major P450 enzyme in normal human blood monocytes [18] and *CYP1B1 *is also present in human lung and lung-derived cell lines [19].

Monocytes, macrophages and epithelial cells are affected by particles. *CYP1B1 *is involved in both, detoxification as well as metabolic activation of xenobiotics [12]. Thus it is important to address the question of whether and in what way particles affect *CYP1B1*. This study is aimed on the effects of carbon particles on the expression of *CYP1B1 *in monocytes/macrophages and bronchial epithelial cells and we demonstrate a pronounced down-regulation of mRNA expression and protein level of this important extrahepatic enzyme.

## Results

### Effect of particle exposure on *CYP1B1 *mRNA expression

In earlier experiments using gene expression arrays, we noted a decreased expression of *CYP1B1 *mRNA in monocyte-derived macrophages (MDM) of patients with COPD (; accession number GSE8608). Since exposure of particles plays a major role in the etiology of this disease, we studied the effect of particles on cells of the monocyte/macrophage lineage. To cover a wider range of particle materials (chemistry) and their physical properties (size, surface structure) and because of economical reasons (cost-effectiveness) we initially used a mixture of both particles, ultrafine P90 (mean size 90 nm) and fine TiO_2 _(mean size 200 nm). We analyzed the effect of this mixture on CD14++ monocytes after 3h of exposure.

**Figure 1:**
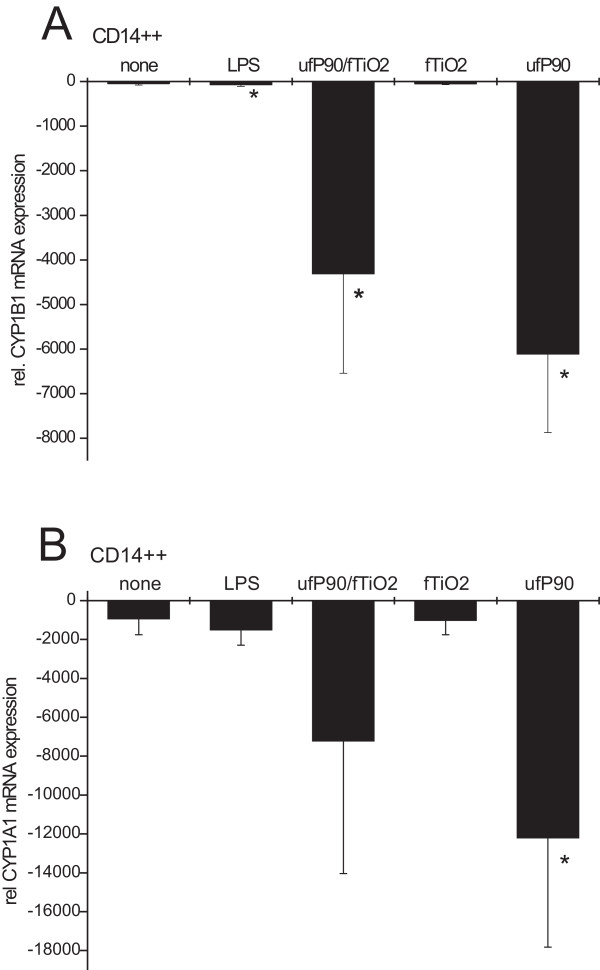
**A) Effect of LPS, ultrafine P90, and fine TiO_2 _on *CYP1B1 *mRNA levels in CD14++ monocytes**. Cells were purified from PBMC of healthy donors by MACS separation. CD14++ cells remained untreated (none) or were stimulated with LPS (10 ng/ml), with particle-mix of ultrafine P90 and fine TiO_2 _(each with 32 μg/ml) or with each particle separately for 3 h. Cells were lysed and mRNA levels were determined by RT-PCR. Data were normalized to levels of the house keeping gene *α-enolase*. (n = 3 incubations from different donors, mean ± S.D.; * p < 0.05 compared to untreated controls). **B) **Down-regulation of *CYP1A1 *mRNA in monocytes. CD14++ cells remained untreated (none) or were stimulated with LPS (10 ng/ml), with particle-mix of ultrafine P90 and fine TiO_2 _(each with 32 μg/ml) or with each particle separately for 3 h. Cells were lysed and mRNA levels were determined by RT-PCR. (n = 3 incubations from different donors, mean ± S.D.; * p < 0.05 compared to untreated controls).

Expression levels were calculated relative to corresponding mRNA level for *α-enolase *in the same sample, such that negative values indicate a transcript prevalence that is less than that of *α-enolase *in the same cell population. As shown in Fig. 1A, incubation of CD14++ monocytes with this mixture of ultrafine P90 and fine TiO_2 _resulted in a pronounced decrease of *CYP1B1 *mRNA transcripts (-4,308 ± 2,231) reflecting a 95-fold reduction compared to untreated cells (-45 ± 37). LPS showed a minor effect with respect to *CYP1B1 *expression (-68 ± 41).

We then addressed the question which of the two types of particles in the mixture caused the down-regulation of *CYP1B1 *mRNA in CD14++ monocytes. As shown in Fig. 1A the decrease of *CYP1B1 *transcripts can be attributed to ultrafine P90 alone which reduced expression 136-fold to a level of -6,109 ± 1,759, while the fine TiO_2 _showed no effect (-50 ± 21) compared to untreated cells (-45 ± 37).

Since the down-regulation of *CYP1B1 *is only found in P90 we next asked whether this was due to the ultrafine nature of the particle. We therefore tested ultrafine TiO_2 _but found no activity with an expression level of -34 ± 20 in untreated and of -27 ± 18 in treated PBMC (n = 3; data not shown). Hence low size is not sufficient a feature to explain the effect on *CYP1B1*. Other properties found in P90 and not in TiO_2 _appear to be responsible.

*CYP1A1 *is a cytochrome monoxygenase closely related to *CYP1B1*, so we asked whether expression of this gene is also influenced by particles. Incubation of CD14++ monocytes with ultrafine P90 for 3 h led to a pronounced 13-fold down-regulation of *CYP1A1 *mRNA (-12,204 ± 5,622). No effect of LPS (-1,508 ± 781) or fine TiO_2 _(-1,013 ± 746) was detected when compared to untreated cells (-940 ± 823) (Fig. 1B). These data show that *CYP1A1 *is also affected by ultrafine P90 albeit to a lesser extent compared to *CYP1B1*.

### Exclusion of LPS-contamination of particles

**Figure 2:**
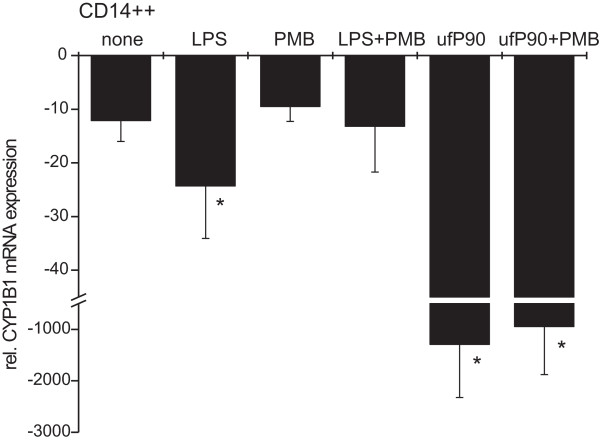
**Particle induced down-regulation of *CYP1B1 *is not due to contaminant LPS**. CD14++ monocytes remained untreated (none) or were stimulated with LPS (10 ng/ml) and ultrafine P90 (32 μg/ml) each with and without 15 min PolymyxinB preincubation to suppress the LPS effect. After incubation for 3 h cells were lysed and mRNA levels were determined by RT-PCR (n = 6 incubations from different donors, mean ± S.D.; * p < 0.05 compared to untreated cells).

A frequent problem in cell biology is LPS-contamination of materials including particles such as those used in our experiments. While in our system LPS alone had a minor effect on its own, we still needed to exclude a contribution of this compound when combined with particles. For this we used Polymyxin B, a compound which is known to inhibit pro-inflammatory signals induced by LPS [20]. Polymyxin B on its own did not alter mRNA expression of *CYP1B1 *(-10 ± 3) compared to untreated cells (-12 ± 4). It did suppress however the moderate LPS-induced decrease of *CYP1B1 *mRNA from -24 ± 10 to -13 ± 9 (p < 0.05). On the other hand the P90-induced down-regulation of expression of *CYP1B1 *mRNA (-1,292 ± 1,031) was not altered by Polymyxin B (-945 ± 935) (not significant, Fig. 2). These findings support the assumption that particles are not contaminated by LPS.

### Dose response and time course of P90-induced *CYP1B1 *mRNA repression

**Figure 3:**
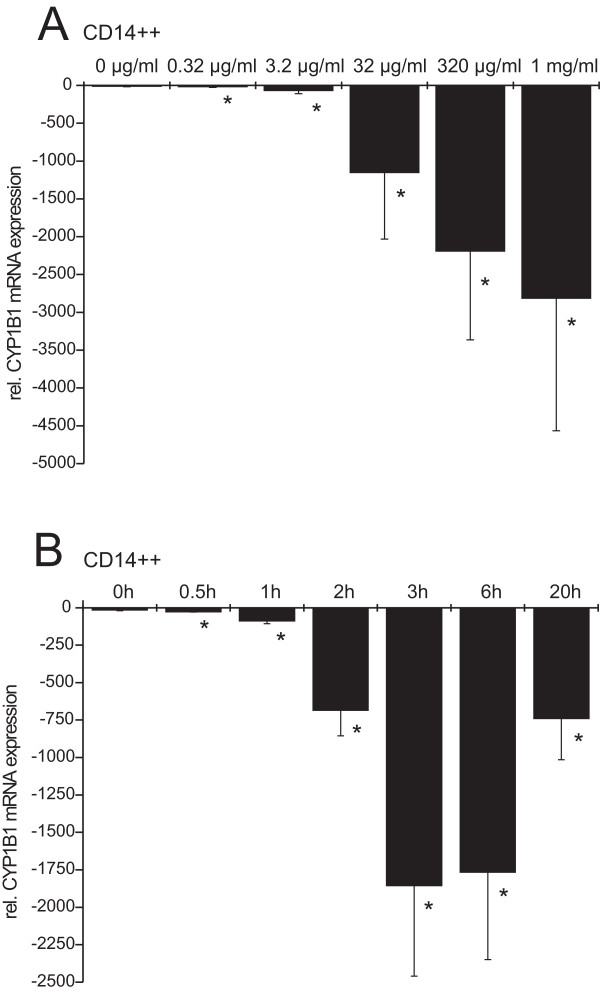
**A) Dose response analysis of ultrafine P90-induced down-regulation of *CYP1B1***. CD14++ cells remained untreated (0 μg/ml) or were stimulated with various doses of 0.32 μg/ml - 1 mg/ml of ultrafine P90 for 3 h. mRNA was recovered and levels of *CYP1B1 *mRNA were determined by RT-PCR (n = 5 incubations from different donors, mean ± S.D.; * p < 0.05 compared to 0 μg/ml). **B) **Time course of ultrafine P90-induced down-regulation of *CYP1B1*. CD14++ monocytes were stimulated for 0, 0.5, 1, 2, 3, 6 and 20 h with ultrafine P90 (32 μg/ml) (n = 3 incubations from different donors, mean ± S.D.; * p < 0.05 compared to 0 h).

To determine optimum dose of ultrafine carbon particles for cytochrome monoxygenase 1B1 mRNA repression we incubated CD14++ monocytes with or without different doses of P90 for 3 h. Subsequently *CYP1B1 *mRNA levels were detected (Fig. 3A). The effects were significant even at low doses, starting with 0.32 μg/ml (-18 ± 8), followed by 3.2 μg/ml (-69 ± 41). A more pronounced decrease of *CYP1B1 *transcripts was seen at 32 μg P90/ml (-1,152 ± 882, 96-fold reduction), at 320 μg P90/ml (2192 ± 1173, 183-fold reduction) and at 1,000 μg/ml (-2,814 ± 1,754, 235-fold reduction). To exclude a toxic effect on cells incubated with the high particle concentrations, a trypan blue viability test was performed. It showed no decrease of cell viability at all particle concentrations. Levels of *α-enolase *mRNA also gave no evidence of loss of viability of these cells (data not shown). For all further experiments we used a particle concentration of 32 μg/ml dose.

Next we analyzed the time course of *CYP1B1 *mRNA repression (Fig. 3B). CD14++ monocytes were incubated with P90 at 32 μg/ml from 0.5 h to 20 h. Repression of *CYP1B1 *was detectable after 0.5 h incubation (-27 ± 2), was more pronounced after 1 h (-89 ± 18) and reached a plateau after 3 and 6 h (-1,856 ± 605 116-fold reduction, and -1,766 ± 584, 110-fold reduction, respectively). After 20 h *CYP1B1 *mRNA levels recovered but were still 46-fold reduced (-740 ± 275) compared to untreated cells. Based on this analysis we used the 3 h time point for all further experiments.

### Effect of particles on *CYP1B1 *and *CYP1A1 *mRNA expression in MDM from healthy donors and COPD patients

**Figure 4:**
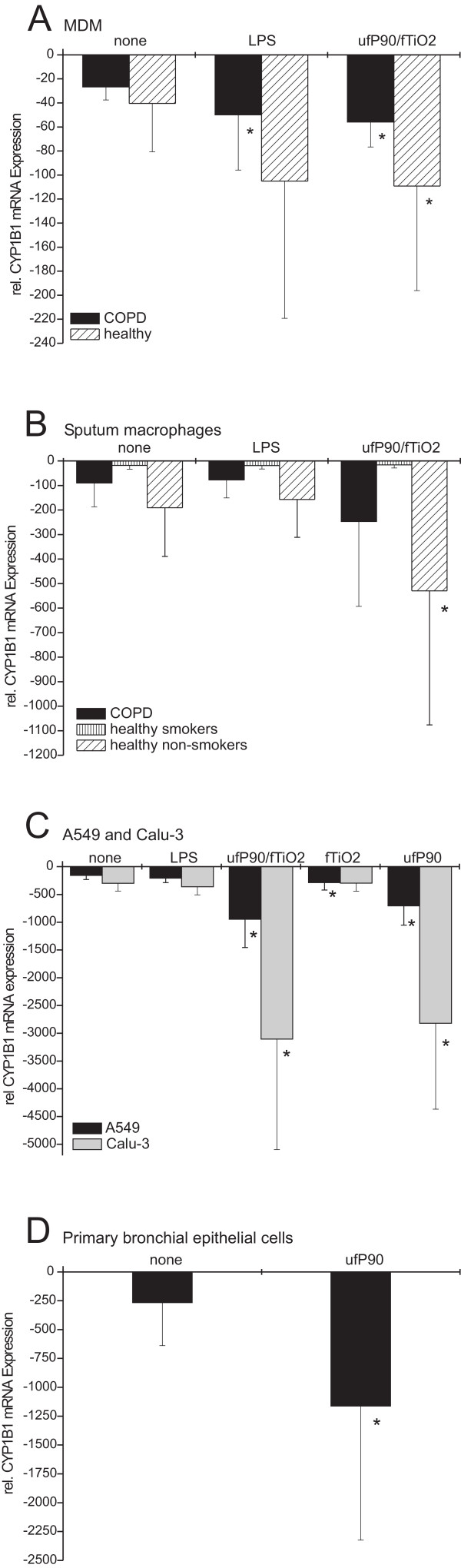
**A) MDM were generated from CD14++ monocytes purified from PBMC by MACS separation followed by 5-day incubation with M-CSF (100 ng/ml)**. Cells remained untreated (none) or were stimulated with LPS (10 ng/ml) or with particle-mix of ultrafine P90 and fine TiO_2 _(each with 32 μg/ml) for 3 h (n = 5 patients with COPD, n = 8 healthy controls, mean ± S.D.; * p < 0.05 compared to untreated controls). **B) **Effect of particles on *CYP1B1 *expression in sputum macrophages of healthy non-smokers, healthy smokers and patients with COPD. Sputum macrophages were purified using RosetteSep to deplete unwanted leukocytes. Cells remained untreated (none) or were stimulated with LPS (10 ng/ml) or with particle-mix of ultrafine P90 and fine TiO_2 _(each with 32 μg/ml) for 3 h (n = 5 non-smokers, n = 4 smokers, n = 7 patients with COPD, mean ± S.D.; * p < 0.05). **C) **Cells remained untreated (none) or were stimulated with LPS (10 ng/ml), with particle mix of ultrafine P90 and fine TiO_2 _(each with 32 μg/ml) or with each particle separately for 22 h (each with 32 μg/ml) (A549 n = 3, Calu-3 n = 4 experiments from different cell passages, mean ± S.D.; * p < 0.05 compared to untreated control). **D) **Effect of ultrafine P90 on *CYP1B1 *mRNA expressin in primary bronchial epithelial cells. Cells were obtained by bronchial brush biopsy. Cells remained untreated (none) or were stimulated with ultrafine P90 (32 μg/ml) for 3 h (n = 7 incubations from different donors, mean ± S.D.; * p < 0.05 compared to untreated control).

MDMs are more mature than monocytes and might show an effect of ultrafine P90 on *CYP1B1 *expression different from what is seen in monocytes. We therefore matured freshly isolated CD14++ monocytes with M-CSF at 100 ng/ml for 5 days and subsequently treated the cells with 32 μg P90/ml for 3 h (Fig. 4A). We used cells from healthy donors and COPD patients to address the question whether these patients have an altered capacity to deal with exogenous particulates.

In untreated MDM of healthy donors a basal mRNA expression of *CYP1B1 *was at -40 ± 40. After LPS stimulation *CYP1B1 *transcript was reduced by factor 2.6 (-105 ± 114, Fig. 4A). Stimulation with the particle-mix of ultrafine P90 and fine TiO_2 _led to a 2.7-fold reduction of *CYP1B1 *mRNA (-109 ± 87, Fig. 4A). Untreated MDM from COPD patients showed a higher level of *CYP1B1 *mRNA (-27 ± 11) and a 2-fold decrease in *CYP1B1 *transcript after incubation with particles (-56 ± 21, Fig. 4A).

Looking at *CYP1A1 *expression in the same MDM we noted a much lower level or constitutive expression compared to *CYP1B1*. In healthy donors *CYP1A1 *mRNA expression was -19,350 ± 17,811, after LPS and particle treatment *1A1 *transcript levels were -84,195 ± 49,677 (4-fold) and -55,720 ± 96,911 (3-fold) respectively. In MDM of COPD patients *1A1 *mRNA levels were -66,840 ± 59,479 in untreated cells and -83,509 ± 66,575 in particle-treated cells (not significant; data not shown).

Taken together MDMs when compared to blood monocytes do show a decrease of cytochrome monoxygenases but the effect appears to be less pronounced with respect to *CYP1B1 *and *CYP1A1*.

### Effect of particles on *CYP1B1 *mRNA expression in sputum macrophages from non-smokers, smokers, and COPD patients

Macrophages in the airways are exposed to inhaled particles and this may impact on *CYP *expression. We therefore isolated macrophages from induced sputum of healthy non-smokers, healthy smokers and COPD patients (6 ex-smokers and one current smoker). In healthy non-smokers particles reduced *CYP1B1 *expression in sputum macrophages 2.8-fold (-530 ± 547) compared to untreated cells (-191 ± 198) (Fig. 4B). In COPD expression of *CYP1B1 *was at -90 ± 97. Treatment of COPD sputum macrophages for 3 h with ufP90/fTiO_2 _led to a 2.7-fold decrease of *CYP1B1 *transcripts (-247 ± 346) compared to 3 h untreated cells. The single currently smoking patient showed the highest mRNA expression of *CYP1B1 *(-22) with no decrease of transcript after ultrafine P90/fine TiO_2 _stimulation (-29).

When looking at healthy smokers we found a very high expression level for *CYP1B1 *(-18 ± 15). Incubation with the particle mixture for 3 h had no effect on *CYP1B1 *transcripts of smokers (-16 ± 13) compared to untreated cells (Fig. 4B). These data show that in smokers sputum macrophages are refractory to the action of particles.

### Effect of particles on *CYP1B1 *mRNA expression in human epithelial cells

Inhaled particles also deposit on alveolar epithelial cells. We thus investigated the effect of ultrafine P90 and fine TiO_2 _on *CYP1B1 *mRNA expression in the human alveolar epithelial cell line A549 and a bronchial epithelial cell line Calu-3. In these experiments we used an incubation time of 22 h as a preliminary time point, which afterwards was optimized depending on cell type (primary cells or cell line) and read-out (transcript or protein analysis).

Compared to untreated A549 cells (-158 ± 73) *CYP1B1 *mRNA levels were decreased after 22 h incubation with P90 by factor 4 (-707 ± 347, p < 0.05) (Fig. 4C). LPS (-206 ± 81) treatment shows no effect, whereas a slight effect was observed with fine TiO_2 _(-287 ± 134) treatment. In cells incubated with both ultrafine P90 and fine TiO_2_, *CYP1B1 *transcript levels were decreased to -948 ± 507.

In the bronchial epithelial cell line Calu-3 the expression of *CYP1B1 *in untreated cells was 301 ± 140 (Fig 4C). Ultrafine P90- (-2,820 ± 1,546) and ultrafine P90/fine TiO_2_-treated cells (-3,106 ± 1,988) showed a strong 10-fold decrease of *CYP1B1 *mRNA. LPS (-360 ± 147) and fine TiO_2 _(-297 ± 144) showed no effect.

To confirm the results of epithelial cell lines we investigated the effects of P90 on *CYP1B1 *mRNA expression in primary bronchial epithelial cells. Cells were obtained by bronchial brush and treated with and without 32 μg ultrafine P90/ml for 3 h. As shown in Fig. 4D*CYP1B1 *transcript levels were reduced 4-fold in ultrafine P90-treated cells (-1,163 ± 1,161) compared to untreated cells (-267 ± 373). This included two cases with a very strong response (35- and 14-fold). Hence primary epithelial cells can be very sensitive to the action of ultrafine carbon particles.

### Effect of benzo[*a*]pyrene on *CYP1B1 *mRNA expression in human peripheral blood mononuclear cells (PBMC)

Cytochrome P450 enzymes are known to be involved in the metabolism of polycyclic aromatic hydrocarbons (PAH). Benzo[*a*]pyrene (BaP), a carcinogenic representative of this class of compounds, strongly up-regulated *CYP1B1 *mRNA levels in PBMC. Time course experiments showed an increase in transcripts from -88 ± 20 in untreated cells to -6 ± 2 in cells after 3 h, and -4 ± 2 after 6 h incubation with BaP (data not shown).

**Figure 5:**
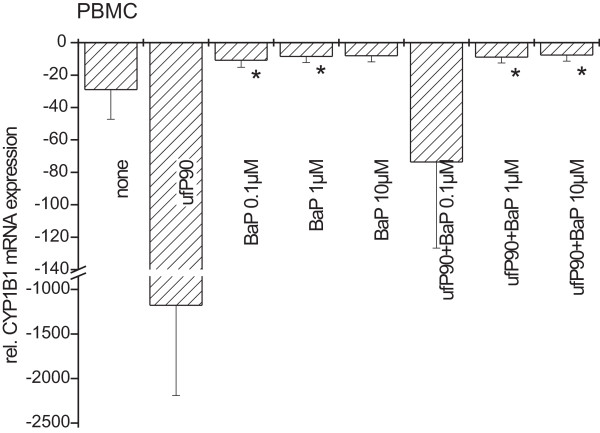
**Interference between ultrafine P90 and benzo[*a*]pyrene (BaP) in *CYP1B1 *induction in PBMC**. Cells remained untreated or pre-stimulated with ultrafine P90 (32 μg/ml) for 30 min. Subsequently cells were stimulated with liposomes containing three different concentrations of BaP (0.1 μM, 1 μM, and 10 μM) and incubated for 3 h. (n = 7 incubations from different donors, mean ± S.D.; * p < 0.05 compared to untreated control).

To investigate whether BaP is capable of overcoming the repression of *CYP1B1 *induced by P90, PBMC were either pre-incubated with ultrafine P90 particles (32 μg/ml) or remained untreated. After 30 min of incubation, BaP-containing liposomes were added in different concentrations (0.1 to 10 μM) (Fig. 5).

*CYP1B1 *mRNA in untreated cells (-29 ± 18) was decreased by ultrafine P90 treatment (-1,178 ± 1,011), on the other hand BaP induced this gene (0.1 μM: -11 ± 4.2; 1 μM: -8.5 ± 3.7; 10 μM: -8.1 ± 3.8). While at high BaP concentrations (1 and 10 μM), ultrafine P90 could not reduce *CYP1B1 *mRNA, it was able to suppress the transcripts by factor 7 at 0.1 μM BaP (p < 0.05).

### Effect of P90 on CYP1B1 protein levels in Calu-3

**Figure 6:**
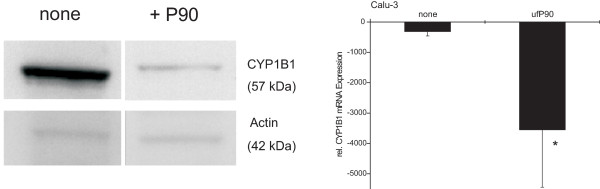
**Western blot analysis of ultrafine P90 effect on CYP1B1 protein in the human lung epithelial cell line Calu-3**. Calu-3 cells were treated 32 h with or without P90 (32 μg/ml). Microsomal protein was isolated and the 57 kDa CYP1B1 protein was detected by a rabbit polyclonal antibody (CYP1B11-A, Alpha Diagnostic). Shown is a representative experiment out of three independent experiments. The right diagram shows the average *CYP1B1 *mRNA expression in Calu-3 cells after 32 h P90 treatment (n = 3, mean ± S.D.; * p < 0.05 compared to untreated cells).

To investigate CYP1B1 protein levels we performed Western blot analysis with isolated microsomal protein of Calu-3 cells. In the blot shown in Fig. 6, the densitometric reading for CYP1B1 protein in Calu-3 decreased from 3,036,276 AU (untreated cells, 32 h) to 373,799 AU after ultrafine P90-treatment for 32 h. In average of 3 experiments CYP1B1 protein level in untreated cells was 2,721,541 ± 379,059 AU and P90 treatment reduced this to 342,109 ± 46,303 AU (Fig. 6). *CYP1B1 *mRNA levels analyzed at the same time point (32 h) and in the same cells used for Western blotting showed a decrease of transcript for ultrafine P90-treated cells (-314 ± 144) compared to untreated cells (-3,551 ± 1,889; p < 0.05) (Fig. 6, right panel).

These data show that P90 will lead to a pronounced reduction of CYP not only at the mRNA but also at the protein level.

## Discussion

The data of this study show that P90 causes a strong down-regulating effect on the *CYP1B1 *expression. Cytochrome P450 monooxygenases are involved in detoxification and toxification of xenobiotic substances. Toxification is caused by metabolic transformation of non or less toxic precursors into reactive intermediates. Cells may thus be influenced by two mechanisms/modes of action, they may either be protected or they may become more susceptible to other inhaled substances by exposure to ultrafine P90.

The strongest down-regulation of *CYP1B1 *expression after stimulation with particle mix of ultrafine P90 and fine TiO_2 _was observed in monocytes (60-fold, Fig. 1A). It is unclear at this point why *CYP1B1 *is so much more sensitive to P90 effects than *CYP1A1 *(Fig. 1B). Whether this is determined at the promoter level needs to be addressed in the future. As P90 was identified as the active component in the particle mix (Fig. 1A) we addressed two questions.

The first question was, whether ultrafine TiO_2 _particles were capable to show such strong down-regulation of *CYP1B1 *mRNA expression as seen with ultrafine P90. To study if the strong P90 effect is triggered by its ultrafine nature (12 nm in diameter, specific surface area of 300 m^2^/g), we incubated cells with ultrafine TiO_2 _with a diameter of 20 nm and a specific surface area of 48 m^2^/g [5]. Neither fine nor ultrafine TiO_2 _treatment did alter *CYP1B1 *mRNA expression. Beck-Speier et al. have shown a highly significant correlation between the PGE_2_/TXB_2 _formation and the specific particle surface area but not the mass concentration [21]. The smaller surface area of ultrafine TiO_2 _could be an explanation for the weaker effect of ultrafine TiO_2 _(48 m^2^/g) compared to ultrafine P90 (300 m^2^/g). Also the chemical composition or surface structure of the particles may contribute to their reactivity towards various cell types. Dick et al. [22] found for ultrafine TiO_2 _and ultrafine P90 purchased from the same manufacturer and with the same composition and surface area than those used herein, different effects in mice when instilled into the lungs. Ultrafine P90 exhibited a higher effect in influx of neutrophile granulocytes into the lungs, higher membrane damage (causes release of G-glutamyl transferase), and higher levels of macrophage inflammatory protein 2 (MIP-2) in the lavage fluid 48 h after instillation than for ultrafine TiO_2_.

Secondly we addressed the question, whether the observed effect was caused by an LPS contamination of P90. LPS contamination can be excluded by neutralizing LPS with Polymyxin B. Our experiments with Polymyxin B showed clearly that the down-regulation of *CYP1B1 *is caused by ultrafine P90 and not by a potential endotoxin contamination of the carbon black particles (Fig. 2). The LPS-mediated reducing effect on *CYP1B1 *expression could be abolished by Polymyxin B, whereas the P90 effect was not altered by Polymyxin B treatment.

With increasing concentrations of ultrafine P90 the decrease of *CYP1B1 *expression becomes stronger. Each of the observed effects was significant. Therefore the P90 concentration of 32 μg/ml was used for all further experiments. The concentration of 32 μg/ml is in accordance with environmentally relevant particle concentration. Higher concentrations do not reflect realistic physiological conditions [23].

We confirmed the reduced *CYP1B1 *mRNA-expression in additional cell types. In MDM (Fig. 4A) we observed a 2.7-fold decrease of *CYP1B1 *after particle treatment. In sputum macrophages of healthy non smokers (Fig. 4B) we showed a 4-fold and of COPD patients a 3-fold reduction of *CYP1B1*. One of the COPD patients currently smoked and showed a high level of *CYP1B1 *mRNA in untreated sputum macrophages that was not affected after ultrafine P90 treatment. Additionally in healthy smokers no effect of particle treatment on *CYP1B1 *transcript level was detected. This may be due to the high concentration of organic compounds, e.g. polycyclic aromatic hydrocarbons (PAH), in cigarette smoke which in turn induced *CYP1B1 *mRNA expression in a competitive manner. A competitional behavior between induction of *CYP1B1 *mRNA by benzo[*a*]pyrene (BaP), a carcinogenic constituent of tobacco smoke [24], and decrease of *CYP1B1 *transcript by ultrafine P90 was clearly shown herein (Fig. 5). Also these findings may suggest that monocytes become less sensitive to ultrafine P90 treatment during maturation, maybe because of a stronger signal transduction or better uptake of particles in monocytes. In parallel to the lower response to particles, sputum macrophages showed no response to LPS with respect to down-regulation of *CYP1B1*.

Epithelial cells are also affected by particle exposure. In epithelial cell lines (Fig. 4C) we observed significant down-regulation of *CYP1B1 *after ultrafine P90 stimulation. Primary epithelial cells obtained by bronchial brush (Fig. 4D) showed in average a 4-fold reduction of *CYP1B1 *mRNA with strong reductions (35- and 14-fold) in two samples. To exclude a leukocyte contamination we analyzed the cells by flow cytometry and microscopic cell differentiation. The stronger effects seen in two of the seven samples may be due to the inter-individual variation or may be due to the different clinical conditions and medications. Taken together there was no apparent clinical feature (diagnosis, medication, smoking status) to explain the higher responses of bronchial epithelial cells in the two cases.

To confirm the *CYP1B1 *mRNA down-regulation on protein level, we isolated the microsomal fraction, because CYP1B1 is bound to the endoplasmatic membrane. It was not possible to isolate sufficient numbers of primary cells in order to obtain enough protein for Western blotting. Therefore for Western blot we used the bronchial epithelial cell line Calu-3 as a model cell line because they also show a decrease in *CYP1B1 *transcripts at mRNA level after ultrafine P90 exposure. The strongest reduction of *CYP1B1 *mRNA was seen after 22 h, therefore we incubated Calu-3 cell with P90 for 32 h to investigate protein expression. Densitometric analysis of the Western blots confirmed a reduction of CYP1B1 protein following P90 treatment (Fig. 6). We assume that also in monocytes and macrophages CYP1B1 protein will be decreased substantially, similar to the mRNA reduction in these cells.

When alveolar macrophages, monocytes, and airway epithelial cells are exposed to particles, they are phagocytized by these cell types [25,26] and can subsequently interfere with gene expression or cell functions. For cells of the monocytic lineage, one possibility is a transport mechanism of particles via the macrophage receptor with collagenous structure (MARCO). In a study of Kanno et al. an association between the uptake of fine and ultrafine particles and MARCO was shown [27]. In our study we blocked the MARCO receptor with antibodies. The reduction of *CYP1B1 *transcripts after ultrafine P90 treatment was not altered by blocking antibodies compared to isotype controls (data not shown). Accordingly the transport mechanism of ultrafine P90 particles appears not to be based on the MARCO receptor and the mechanism involved is still elusive.

Transcription of *CYP1B1 *and *CYP1A1 *genes is regulated by the interaction between PAH and the cytosolic arylhydrocarbon receptor. Dioxin is the most potent agonist [17], but also BaP can activate the receptor complex [28]. Cigarette smoke and diesel soot contain a huge amount of PAH adsorbed to particles [29,30]. Herein we showed a BaP-mediated induction of *CYP1B1 *in a concentration-dependent manner (Fig. 5). Simultaneously treatment with ultrafine P90 attenuates the *CYP1B1 *induction. The attenuation may be less pronounced with low concentration of BaP (0.1 μM) because of absorption effects of BaP onto P90 particles, which reduce the amount of bioavailable BaP. Overall these findings indicate a competing behaviour of BaP and P90. In case of low BaP concentrations (0.1 μM), P90 still reduces *CYP1B1 *expression and this may affect its detoxifying/toxifying activity.

Besides *CYP*-gene products, myeloperoxidase (MPO) was reported to play an important role in metabolic activation of chemical carcinogens [31]. MPO catalyzes the conversion of H_2_O_2 _into hypochlorous acid (HOCl), which is a very strong oxidant and which in turn may be responsible for the metabolic activation of inhaled chemicals or organic compounds on inhaled particles. In our investigations we screened CD14++ monocytes for expression of *MPO *on mRNA level after exposure to particles (Printex 90 and fine TiO_2_). Compared to resting monocytes (-641), particle incubation (3 h) decreased *MPO *mRNA expression 4-fold (-2,662; not significant) (data not shown). This result indicates that particles alter the expression of a broader variety of enzymes which are involved in inflammation and the metabolism of xenobiotics. Little is known about the mechanisms and specificity of this interaction and further investigations addressing this question will be needed.

Also cytokines modulate *CYP*-gene expression. P90-induced transcription factor NF-κB in alveolar macrophages leads to expression of pro-inflammatory cytokines like TNF, IL-6, and IL-1β [32]. TNF in turn increases the expression of *CYP1B1 *[33]. Umannová et al. report of TNF-induced increase of *CYP1B1 *mRNA expression and simultaneously suppression of BaP-induced *CYP1A1 *expression [34]. This dysregulation was found to be associated with an enhanced formation of DNA adducts and enhanced genotoxic effects.

*CYP*-expression is also regulated by *aryl hydrocarbon receptor (AhR) *and *AhR nuclear translocator (Arnt)*. Wu et al. [35] discussed the reduction of *AhR*- and *Arnt*-expression as the underlying mechanism for TNF-induced decrease of *CYP1A2 *expression after LPS-treatment. In our system no influence of particles was detected on *AhR *and *Arnt *on transcriptional level (data not shown).

While a lot is known about induction of *CYP*-genes little information is available on suppression of these genes by particles. We show herein an unexpected and pronounced reduction of *CYP *expression in various cell types. The reduction of CYP protein may lead to disruption of various cellular functions: pathophysiological response to stress signals (toxic substances or inflammation), an adaptive homeostatic response (controlled generation of reactive oxygen species) or a part of tightly regulated physiological pathway (production of bile acid) [36]. Inflammatory mediators can be responsible for a reduction of *CYP *mRNA [37]. Ke et al. reported that one mechanistic explanation for LPS and cytokine-mediated *CYP *reduction may be an interaction with NFκB [38].

The reduction of *CYP1B1 *after ultrafine P90 treatment could be a form of protective mechanism concerning oxidative stress. Reactive oxygen species (ROS) generated by *CYP*-catalyzed metabolisms can cause oxidative stress in cells [39]. Reduced CYP1B1 may prevent further DNA damage being caused by ROS. Furthermore, decreased availability of CYP1B1 enzyme leads to a lesser activation of PAH to reactive metabolites which otherwise could cause DNA damage and cancer development [12]. On the other hand *CYP1B1 *and *CYP1A1 *are also important for detoxifying various xenobiotics. A reduced expression of these cytochromes in monocytes/macrophages and lung epithelial cells induced by inhaled carbon particles may lead to increased damage by xenobiotics because of the lack of its detoxifying character. Further investigations will be required to elucidate by which mechanism the particles affect the *CYP1B1 *expression.

## Methods

### Cell lines and culture medium

The epithelial cell lines A549 [40] and Calu-3 [41] were cultured in Dulbecco's minimal essential medium NUT mix F12 (21331-020, Invitrogen, Karlsruhe, Germany), containing 10% FCS (S0115, Biochrom, Berlin, Germany), L-glutamine 2 mM (15140-114, Invitrogen, Karlsruhe, Germany), and penicillin 200 U/ml/streptomycin 200 μg/ml (15140-114, Invitrogen, Karlsruhe, Germany).

Primary human cells were cultured in RPMI 1640 medium (F1415, Biochrom, Berlin, Germany), supplemented with OPI (oxaloacetate, pyruvate, insulin; O-5003, Sigma, Taufkirchen, Germany), L-glutamine 2 mM (15140-114, Invitrogen, Karlsruhe, Germany), penicillin 200 U/ml/streptomycin 200 μg/ml (15140-114, Invitrogen, Karlsruhe, Germany), and 1× nonessential amino acids (11140-35, Invitrogen, Karlsruhe, Germany). Before addition of 10% FCS (S0115, Biochrom, Berlin, Germany), the supplemented medium was ultrafiltered through a Gambro 2000 column (Gambro, Hechingen, Germany) in order to remove any inadvertent LPS contamination.

### Donors of primary human cells

Primary human cells were obtained from healthy human volunteers (non-smoker and smoker), from patients with COPD, or from patients with other lung diseases (Table 1). Written informed consent was obtained from each individual. The protocol was approved by the Ethics Committee of the Medical School of the Ludwigs-Maximilians-University (Munich, Germany).

**Table 1:** Characteristics of patients and volunteers from which primary human cells were obtained.

	**number**	**sex**	**age**	**disease and stage**
normal healthy	23	12× m, 11× f	42.8 ± 15.4	n.a.
healthy smokers	6	4× m, 2× f	54.5 ± 10.6	n.a.
COPD patients	12	10× m, 2× f	63.9 ± 10.5	4× II, 3× III, 5× IV (Gold)
others (brush biopsies)	7	5× m, 1× f, 1× anonymized*	65.8 ± 11.7*	adenocarcinoma, MALT-lymphoma, UIP, sarcoidosis (Gold II), COPD (Gold III)*

(*: samples from one patient who underwent brush biopsy was anonymized; f: female; m: male; MALT: mucosa-associated lymphoid tissue; n.a.: not applicable; UIP: usual interstitial pneumonia).

### Isolation of peripheral blood mononuclear cells (PBMC), enrichment of CD14++ monocytes, and generation of monocyte-derived macrophages (MDM)

Human PBMC were isolated from heparinized (10 U/ml) blood by density gradient centrifugation (Lymphoprep, 1114545, 1.077 g/ml, Oslo, Norway). Cells were directly used for subsequent isolation of monocytes and cultured under LPS-free conditions.

For cell enrichment the MACS magnetic separation technique was used (all columns and reagents from Miltenyi Biotec, Bergisch-Gladbach, Germany). For isolation of CD14++ monocytes, PBMC were in a first step depleted of CD16-positive cells. For this, a total of 20 × 10^6 ^cells were resuspended in 80 μl of PBS containing 25 μl of Anti-CD16 microbeads (130-045-701). After incubation for 30 min at 4°C cells were washed and resuspended in 1.5 ml PBS and this was loaded onto a LD column (120-000-497) that was positioned in a MidiMACS magnet (130-042-302). Nonadherent cells were recovered and used for enrichment of CD14++ cells. For this, anti-CD14 microbeads (130-050-201) was diluted 1:5 in PBS and added to the cells to a final volume of 100 μl. After incubation for 30 min at 4°C cells were washed and resuspended in 1.5 ml PBS and this was loaded onto a LS column (120-000-475). The column was washed five times with 2 ml PBS each. Cells were recovered from the column by flushing the column five times with 2 ml PBS using a plunger. CD14++ cells were washed and resuspended in supplemented RPMI 1640 medium (mentioned above).

To determine purity of the CD14++ monocytes, a sample was stained with fluorescein isothiocyanate (FITC)-labeled anti-CD14 antibody (My4-FITC, 6603511, Coulter, Krefeld, Germany) and phycoerythrin (PE)-labeled anti-CD16 antibody (Leu11c-PE, Becton-Dickinson, Heidelberg, Germany) and measured by flow cytometry. CD14++ monocytes with a purity of 92% or higher were used.

For generation of MDM, CD14++-monocytes were cultivated in a low-attachment 24-well plate (3473, Costar, Wiesbaden) and cultured for 5 days with 100 ng/ml M-CSF (rhM-CSF, kindly provided by Genetics Institute, Cambridge, Massachusetts, USA).

### Sputum induction and processing

Sputum macrophages were collected from healthy human volunteers and from patients with COPD after informed consent. The subjects were instructed to mouthwash with water. Sputum induction was performed by stepwise inhalations for 10 min each with increasing concentrations of sodium chloride (0.9%, 3%, and 5% in healthy individuals and 0.9% and 3% in COPD patients), nebulized by an ultrasonic nebulizer (Multisonic LS 290, Schill, Probstzella, Germany). After inhalation individuals were encouraged to cough deeply. Sputum was coughed into petri dishes (d = 13.5 cm) and processed immediately.

To homogenize the sputum samples by cleavage of disulphide bonds of mucin glycoproteins, four volume parts of sputolysin reagent (560000, Calbiochem, Bad Soden, Germany) containing 6.5 mM dithiothreitol and 100 mM phosphate buffer (pH 7.0) were added. After vortexing briefly, the mixture was incubated at 37°C and vortexed every 10 min until the sputum was homogenized, in total no longer than 50 min. The sputum samples were diluted with 1 volume PBS (Gibco, Karlsruhe, Germany) and filtered consecutively through a 100 μm and a 40 μm mesh filter (352360 and 352340, Becton Dickinson, Heidelberg, Germany). Cells were then pelleted by centrifugation for 10 min at 400 × g and 4°C and resuspended in 3 ml PBS. 10 μl packed erythrocytes and 50 μl RosetteSep Human Monocyte Enrichment Cocktail (15068, CellSystems, St. Katharinen, Germany) were added, which crosslinks unwanted cells to multiple red blood cells, forming immunorosettes. After 20 min incubation at room temperature the sample was layered on the top of lymphoprep and centrifuged 30 min at 800 × g. The enriched macrophages were harvested from the plasma interface.

### Brush biopsy of airway epithelial cells

Patients who underwent diagnosic bronchoscopy were brush biopsied with a sterile single-sheated nylon cytology brush (BC-202D-5010, Olympus EndoTherapy, Hamburg, Germany). Four consecutive brushings from an intrabronchial area were taken from the proximal part of one of the main bronchi. Epithelial cells were harvested from the brush by agitating it in 5 ml of RMPI 1640 medium. After centrifugation cells were lysed in TRI reagent (T-9424, Sigma, Taufkirchen, Germany) and RT-PCR analysis was performed (see below).

### Treatment of cells

For stimulation with lipopolysaccharide (LPS; L-6261, Sigma, Taufkirchen, Germany) blood cells were incubated with 10 ng/ml, sputum macrophages with 1 μg/ml for 3 h each, or remained untreated as control. For particle-stimulation with ultrafine P90 (14 nm in diameter, Degussa, Frankfurt, Germany) and fine TiO_2 _(220 nm in diameter, Degussa, Frankfurt, Germany), cells were incubated for 3 h with 32 μg/ml (each particle) or remained untreated as control. For particle concentration experiments CD14++ monocytes were incubated with ultrafine P90 for 3 h at doses of 0.32 μg/ml - 1 mg/ml and 37°C. Using the trypan blue exclusion assay, cell viability was not found to be altered by ultrafine P90-treatment at any particle concentration used herein. For time course experiments cells were incubated for different periods of time (0.5 to 20 h) with P90 (32 μg/ml). For stimulation with BaP (12780, Fluka, Taufkirchen, Germany) a 1:50 dilution of BaP-containing liposomes was used. For preparation of liposomes di-oleyl-phosphoserine (OOPS) and palmitoyloleyl-phosphocholine (POPC) were dissolved at an OOPS: POPC ratio of 0.43 in chloroform. BaP in chloroform was added at different concentrations. Liposomes were generated by evaporating organic solvent followed by adding aqueous buffer. Resulting liposomes can be stored up to 4 weeks at 4°C.

Experiments were performed at least in triplicates, using primary cells from different donors, or cell lines from different passage numbers.

### Total RNA isolation and RT-PCR

Polymerase chain reaction (PCR) was performed according to the method of Wang and colleagues [42]. Total RNA was extracted from cells by using TRI Reagent (T-9424, Sigma, Taufkirchen, Germany) according to the manufacturer's instruction. In brief cells were lysed in 200 μl TRI Reagent and 15 μg tRNA (109517, Roche, Mannheim, Germany) as carrier were added per sample. After isolation, the RNA was reverse transcribed with oligo(dT) as primer.

Using the LightCycler system (Roche Diagnostics, Mannheim, Germany) according to the manufacturer's instruction, semi-quantitative PCR were performed with the following primers:

*CYP1A1*; 5' primer: 5'-TCT TTC TCT TCC TGG CTA TCC T-3'

3' primer: 5'-CTG TCT CTT CCC TTC ACT CTT G

*CYP1B1*; 5' primer: 5'-TGA TGG ACG CCT TTA TCC TCT C-3'

3' primer: 5'-CAT AAA GGA AGG CCA GGA CAT A-3'

*MPO; *5' primer: 5'-TCG GTACCC AGT TCA GGA AGC-3'

3' primer: 5'-CCA GGT TCA ATG CAG GAA GTG T-3'

*α-enolase*; 5' primer: 5'-GTT AGC AAG AAA CTG AAC GTC ACA-3'

3' primer: 5'-TGA AGG ACT TGT ACA GGT CAG-3'

3 μl of cDNA were used for amplification in the SYBR Green format using the LightCycler-FastStart DNA Master SYBR Green I kit from Roche (2239264, Mannheim, Germany). For PCR analysis, the LightCycler system offers the advantage of fast and real-time measurement of fluorescent signals during amplification. The SYBR Green dye binds specifically to the minor groove of double stranded DNA. Fluorescence intensity is measured after each amplification cycle. During PCR, a doubling of template molecules occurs in each cycle only during the log-linear phase. Melting curves have been performed after amplification to ensure that primer dimers did not contribute to the fluorescence intensity of the specific PCR-product. Amplificates were run out on a 2% agarose gel and bands were observed on the expected molecular weight. As an internal control the housekeeping gene *α-enolase *was amplified.

For analyzing the LightCycler data all samples (tested gene and housekeeping gene) are processed in the LightCycler software with the same settings (e.g. same thresholds). Cycle number of the housekeeping gene was subtracted from the corresponding housekeeping gene and its absolute value was subsequently calculated to the power of 2. Genes with a higher cycle number than the corresponding housekeeping gene were plotted to the negative scale.

### Western blot

For Western blot analysis the microsomal fraction was prepared. After a 32 h incubation with and without 32 μg P90/ml Calu-3 cells were harvested and the pellet was resuspended in 4 volumes of hypo-osmolar buffer A (10 mM HEPES pH 7.9, 10 mM KCl, 1.5 mM MgCl_2_) in presence of a protease inhibitor mixture consisting of 10 μg/ml aprotinin (A-6279, Sigma), 1 mM PMSF (P-7626, Sigma), 40 μg/ml leupeptin-propionyl (L-3402, Sigma), 20 μg/ml leupeptin-acetate (L-2023, Sigma), 20 μg/ml antipain (A-6191, Sigma), 20 μg/ml pepstatin A (P-4265, Sigma), 400 μM ALLN (A-6185, Sigma), and 2 mM DTT (19474, Merck). The lysates were incubated for 10 min on ice prior to sonication (8 × 10 sec) and then centrifuged at 3,000 × g for 10 min at 4°C. The resulting supernatant was centrifuged at first at 12,000 × g for 12 min at 4°C and then at 25,000 × g for 2 h at 4°C to yield the microsomal fraction. The microsomal pellet was solubilized in 30 μl of buffer D (20 mM HEPES pH 7.9, 20% Glycerol, 0.1 M KCl, 0.05 mM EDTA) in presence of a protease inhibitor mixture and 0.1% SDS and then stored at -80°C.

Protein concentrations were determined using Bradford reagent. 10 μg of protein were resolved on a 4-12% Novex bis-tris gel (NP0329BOX, Invitrogen, Karlsruhe) and transferred to Hybond-N membranes (LC2001, Invitrogen) using a Novex X-Cell II Mini Cell. Membranes were blocked with blocking buffer (TBS supplemented with 5% fat-free milk powder (1.15363, Merck) and 0.05% Tween20 (2323003, Wasserfuhr, Bonn)) and then reacted with anti CYP1B1 antibody (rabbit polyclonal, CYP1B11-A, Alpha Diagnostic) over night and anti actin (A-2066, Sigma) for 1 h. As a second antibody a peroxidase-conjugated anti rabbit IgG (A0545, Sigma) was used. Bound antibody was detected using the ECL kit (RPN2106, Amersham) and membranes then were exposed to Hyperfilm™ECL (RPN3103, Amersham).

Densitometric analysis was performed using NIH ImageJ software (Version 1.39u, NIH, Bethesda, MD, USA).

### Statistics

For statistical analysis of the data, we used the Student's T-test. Results were considered significant if *p *< 0.05.

## Abbreviations

BaP: benzo[*a*]pyrene; COPD: chronic obstructive pulmonary disease; CYP: Cytochrome P450; f: fine; h: hour; LPS: lipopolysaccharide; MDM: monocyte-derived macrophage; P90: Printex 90; PAH: polycyclic aromatic hydrocarbons; PBMC: peripheral blood mononuclear cells; ROS: reactive oxygen species; uf: ultrafine

## Competing interests

The authors declare that they have no competing interests.

## Authors' contributions

CE was substantially involved in conducting the experiments and drafting the manuscript. MF, FS, AS, and K-WS were in involved in conducting the experiments. LZH is one of the project leaders and was involved in the design of the study. TH is the main project leader, involved in the design of the study, in conducting experiments, and in drafting the manuscript.

All authors read and approved the final manuscript.
